# Subcutaneous leiomyosarcoma in a cynomolgus macaque (*Macaca fascicularis*)

**DOI:** 10.1002/vms3.1321

**Published:** 2024-01-16

**Authors:** Dong‐Ho Lee, Jun Won Mo, Seung‐Bin Yoon, Jeongwoo Kwon, Yu‐Jin Jo, Sang Il Lee, Taeho Kwon, Jungkee Kwon, Ji‐Su Kim

**Affiliations:** ^1^ Primate Resources Center Korea Research Institute of Bioscience and Biotechnology (KRIBB) Jeongeup Jeonbuk Republic of Korea; ^2^ Department of Laboratory Animal Medicine College of Veterinary Medicine Jeonbuk National University Iksan Jeonbuk Republic of Korea

**Keywords:** cynomolgus macaque, leiomyosarcoma, *Macaca*, smooth muscle cells, soft tissue neoplasm

## Abstract

Leiomyosarcoma, a malignant tumour originating from smooth muscle cells, has rarely been documented in non‐human primates. In this case study, a 7‐year‐old female cynomolgus macaque (*Macaca fascicularis*) presented with a rapidly growing mass overlying the left elbow joint. Radiographs indicated the presence of a soft tissue neoplasm without any associated bone involvement. The mass was surgically resected. Histological and immunohistochemical analyses revealed spindle‐shaped cells with eosinophilic cytoplasm that resembled smooth muscle cells, exhibiting positive immunoreactions for vimentin, desmin and smooth muscle actin and a negative reaction for pan‐cytokeratin. This is the first reported case of subcutaneous leiomyosarcoma in a cynomolgus macaque and provides important insights into the incidence and characteristics of this condition in this species.

## Graphical abstract

Graphical Abstract: This report describes a rare case of leiomyosarcoma in the left elbow of a cynomolgus macaque. The study provides important information regarding the incidence and characteristics of this tumour in the species, with a detailed examination through histological and immunohistochemical analyses.

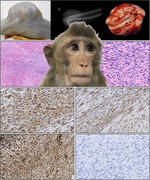

## 1 INTRODUCTION

Leiomyosarcoma is a type of malignant tumour derived from smooth muscle cells; it represents a significant clinical challenge owing to its aggressive behaviour and limited treatment options (Ahuja et al., 2017; Gladdy et al., 2013; Sbaraglia et al., 2021). The tumour is distinguished by wide interlacing fascicles composed of spindle‐shaped or ovoid neoplastic cells (Miettinen, 2016). These neoplastic cells retain many features characteristic of normal smooth muscle cells, such as elongated nuclei and eosinophilic cytoplasm (Weiss et al., 2019). This type of neoplasm appears relatively frequently in the visceral organs, particularly in the gastrointestinal tract and female reproductive system, of humans and most other animals (Birkebak et al., 1996; Cook et al., 2004; George et al., 2018; Meuten, 2020; Weiss et al., 2019).

Despite its relatively high prevalence in the visceral organs of humans and animals, leiomyosarcoma is also known to occur in less common anatomical sites, such as the oral cavity, oesophagus, blood vessels and bladder, as documented in a limited number of studies (Abed et al., 2009; Liu & Mikaelian, 2003; Meuten, 2020; Weiss et al., 2019). Although subcutaneous leiomyosarcoma in animals is rare, it has been reported in some species, such as cows, hamsters, wolves and squirrel monkeys (Bock et al., 2007; Brunnert et al., 1990; de Castro Pires et al., 2017; Hanzaike et al., 1995; Nakamura et al., 2010; Park et al., 2010; Yi et al., 2008).

The cynomolgus macaque (*Macaca fascicularis*), a highly relevant species in both biomedical and zoological research (Gardner & Luciw, 2008; Kaplan & Manuck, 1999; Kaplan et al., 1984; Lin et al., 2009), is known to have relatively few reported tumour cases. Malignancies, such as malignant lymphoma and squamous cell carcinoma, as well as benign tumours, such as leiomyomas and polyps in various organs, have been described in previous reports (Bright et al., 2019; Kaspareit et al., 2007; Schmelting et al., 2011).

Here, we report a case of leiomyosarcoma in the left elbow of a cynomolgus macaque, providing important information regarding the incidence and characteristics of this tumour in the species.

## 2 CASE DESCRIPTION

**FIGURE 1:**
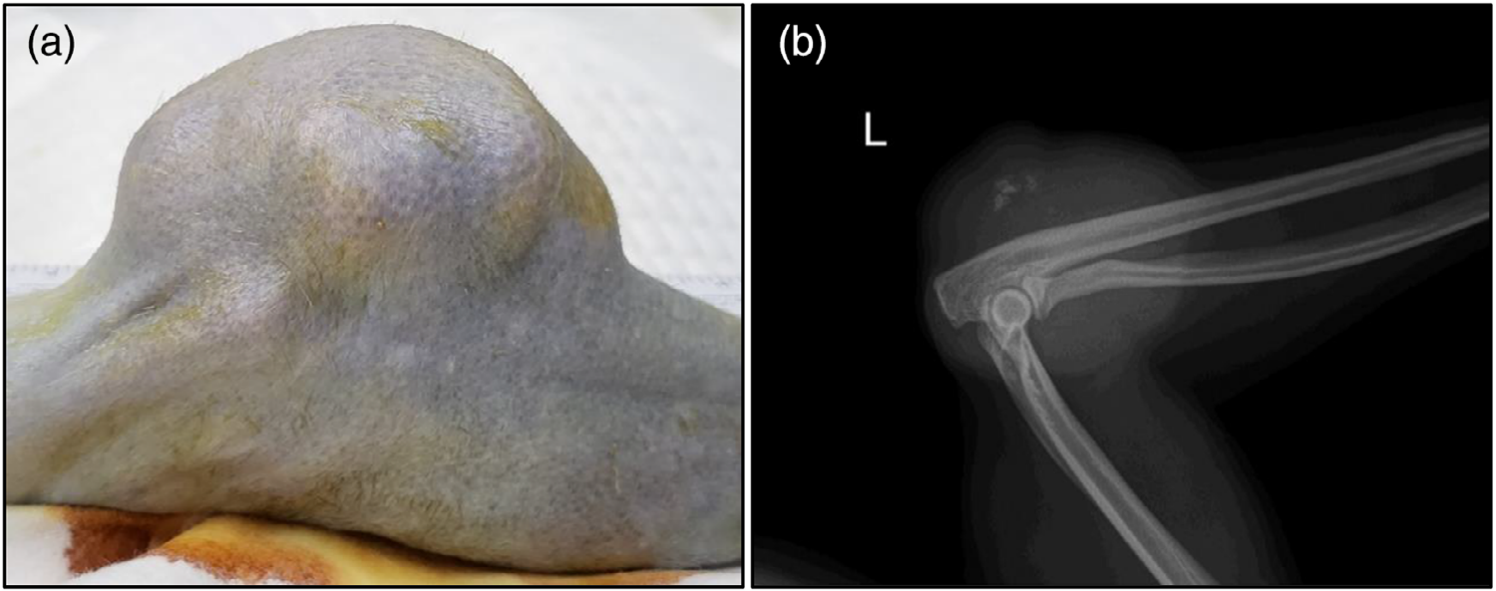
Gross and radiographic findings of leiomyosarcoma: (a) anterior view of the mass on the left elbow joint and (a) radiographic image of the left elbow. The mass did not invade the elbow joint and nearby bones.

A 7‐year‐old female cynomolgus macaque, weighing 4.86 kg, with an abnormality in her left elbow was observed at the Primate Resources Center (Jeongeup, South Korea) (Figure 1A). The macaque had normal food intake, faecal condition and vitality. However, a mass developed over her left elbow joint and rapidly increased in size. No signs of necrosis or ulceration were observed on the skin above the mass. Radiography indicated that the mass had not invaded the elbow joint or nearby bones, and that there was calcification in some portions of the tissue (Figure 1B).

**FIGURE 2:**
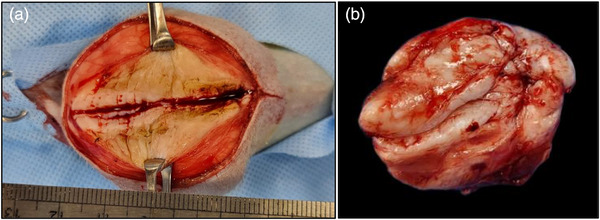
Surgical excision of the subcutaneous mass on the left elbow joint: (a) intraoperative photograph showing the surgical incision of the mass using an electric surgical knife and (b) postoperative photograph of a greyish‐white or white solid mass with haemorrhage in the subcutaneous tissue, which was located medial to the elbow joint.

The mass was surgically resected; it was present in the subcutaneous tissue of the left elbow joint and measured 7.5 × 6.0 × 4.5 cm^3^ (Figure 2A). The mass was in contact with the skeletal muscles but did not infiltrate them. The cut surface of the mass was solid, appeared white or greyish‐white and showed signs of haemorrhage (Figure 2B).

The tissue samples were collected for further analysis. The collected samples were fixed in 10% phosphate‐buffered formalin and sectioned to a thickness of approximately 5 μm for haematoxylin‐eosin staining and immunohistochemical analysis. Immunohistochemistry (IHC) was performed to evaluate the expression of several markers in the tissue samples. Primary mouse monoclonal antibodies were used to detect vimentin (M0725, clone V9, Dako; 1:50), smooth muscle actin (M0851, clone 1A4, Dako; 1:100), desmin (MA1‐06401, clone D9, Thermo Scientific; 1:100) and pan‐cytokeratin (CM011B, clone AE1/AE3, Biocare Medical; 1:70). Following incubation with primary antibodies, the sections were incubated with a mouse anti‐mouse secondary antibody (K4001, Dako) and then stained with diaminobenzidine (K3467, Dako).

**FIGURE 3:**
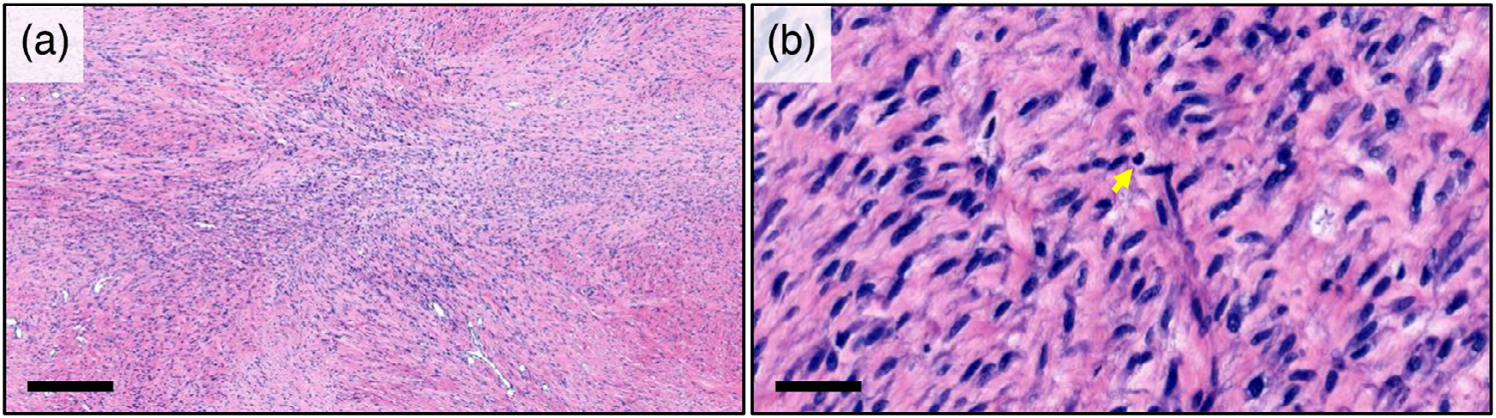
Histopathological analysis of the subcutaneous mass: (a) low‐magnification haematoxylin and eosin (H&E) stained image showing highly cellular neoplastic tissue with pleomorphic cells and an atypical pattern of muscle fibres. Scale, 500 µm. (b) High‐magnification H&E‐stained image showing individual neoplastic cells, ranging from small and round to large and polygonal, with eosinophilic cytoplasm. A rare mitotic figure is indicated by the arrow. Scale, 50 μm.

Histologically, the tumour exhibited extensive proliferation of eosinophilic smooth muscle bundles. The proliferation of tumour cells exhibited partial density, and neoplastic cells were observed interweaving in various directions, occasionally forming a herringbone pattern (Figure 3A). The proliferation area was represented by small‐ to medium‐sized spindle‐shaped cells that formed bundles resembling a smooth muscle. Some of these cells had pleomorphic nuclei, multiple and indistinct nucleoli, and moderate‐to‐abundant eosinophilic cytoplasm (Figure 3B). The mitotic count within a 2.37 mm^2^ field of observation was low, with one mitotic figure. Additionally, focal areas of fibrosis and infiltration of lymphocytic inflammatory cells were observed.

**FIGURE 4:**
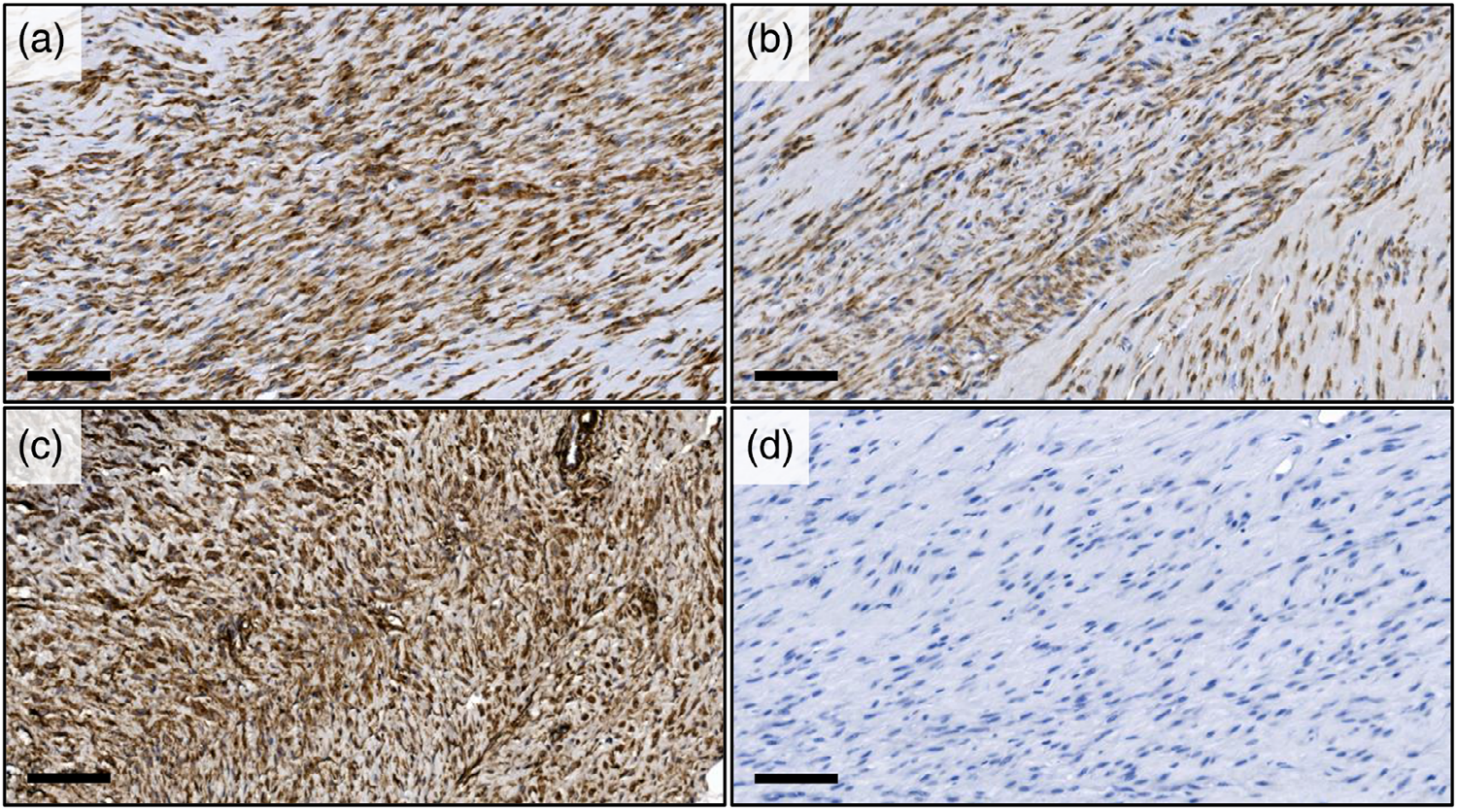
Immunohistochemical staining of the tumour tissue for vimentin, desmin, smooth muscle actin and pan‐cytokeratin: (a) Vimentin immunostaining revealed strong positive staining in neoplastic cells, indicating mesenchymal differentiation; (b) desmin immunostaining demonstrated positive staining in neoplastic cells, indicating muscle differentiation; (c) smooth muscle actin immunostaining exhibited strong positive staining in neoplastic cells, suggesting smooth muscle differentiation; and (d) pan‐cytokeratin immunostaining showing negative staining, indicating the absence of epithelial differentiation. Scale, 100 µm.

Immunohistochemical analysis revealed strong positive staining for vimentin (approximately 50%–70% positive cells with intense reaction) and for smooth muscle actin (greater than 90% positive cells with intense reaction), as well as positive staining for desmin (approximately 30%–50% positive cells with moderate reaction) in neoplastic cells. However, it was negative for pan‐cytokeratin (Figure 4A–D).

One year has elapsed since the resection of the tumour without any confirmed case of recurrence or metastasis to the removal or other sites, indicating the effectiveness of the surgical procedure employed.

## 3 DISCUSSION

Leiomyosarcoma is a malignant mesenchymal tumour characterized by interlacing bundles of smooth muscle fibres (Miettinen, 2016). It predominantly arises in deep soft tissue sites, such as the retroperitoneum; however, its occurrence in superficial soft tissue is uncommon. Based on the origin within superficial soft tissue, leiomyosarcoma can be classified into subcutaneous and cutaneous types (Guillén & Cockerell, 2001; Torres et al., 2011; Wascher & Lee, 1992).

Subcutaneous leiomyosarcomas are rare and account for approximately 4%–6.5% of all soft tissue sarcomas in humans (Jena et al., 2014). Similarly, cases of subcutaneous leiomyosarcoma in animals are rare. In humans, subcutaneous leiomyosarcoma tends to have a high rate of recurrence (37%) and metastasis (21%–62%), which makes the prognosis less favourable (Soares et al., 2021). The situation is similar for animals with leiomyosarcoma, as they generally have a poor prognosis and a low chance of long‐term survival (Cohen et al., 2003; Kapatkin et al., 1992; Patnaik et al., 1986).

Leiomyosarcomas display a broad spectrum of atypia, ranging from well‐ to poorly‐differentiated tumours with variable histologic patterns (Winchester et al., 2014). In previous studies, leiomyosarcoma has exhibited variable mitotic activity, and in addition to cellular anaplasia, even a single observed mitotic figure has been considered indicative of malignancy (Bernstein & Roenigk, 1996; Cook et al., 2004; Demicco et al., 2015; Fauth et al., 2010; Fields & Helwig, 1981; Hornick & Fletcher, 2003; Miettinen, 2016; Winchester et al., 2014).

Diagnosing leiomyosarcoma can be challenging due to its histological similarity with other spindle‐shaped neoplastic cell tumours (Arai et al., 2010; Farcas et al., 2014; Fields & Helwig, 1981; Hamali & Ashrafihelan, 2010; Hayden et al., 1999; Hollmig et al., 2012; Perez et al., 1996; Sotiriou et al., 2020). This similarity can lead to difficulties in distinguishing leiomyosarcoma from other tumours based solely on morphological characteristics. Therefore, IHC, using antibodies that bind to specific proteins in tissue samples to help identify and characterize cells or structures of interest, is crucial for tumour evaluation (Folpe & Cooper, 2007; Schütz et al., 2013; Snowden et al., 2001). The inclusion of IHC in tumour evaluation significantly contributes to enhancing diagnostic accuracy.

In this case, histologically, within certain regions of well‐differentiated neoplastic cells, neoplastic cells displayed nuclear pleomorphism, variable nuclear sizes and rare mitotic figures, meeting the criteria for malignant diagnosis. Immunoreactivity for vimentin, desmin and smooth muscle actin was observed in the neoplastic cells, indicating a mesenchymal and smooth muscle origin (Hollmig et al., 2012; Leader et al., 1987; Painter et al., 2010), which is suggestive of the possibility of leiomyosarcoma. The absence of a positive reaction to pan‐cytokeratin, a marker for tumour cells of epithelial origin (Hollmig et al., 2012; Menz et al., 2022; Painter et al., 2010), further supports the diagnosis of leiomyosarcoma. These findings are also consistent with previous research conducted on both human and animal subjects (Carvalho et al., 2009; Cook et al., 2004; de Castro Pires et al., 2017; Nakamura et al., 2010), thereby ruling out other tumour types, including osteosarcoma, synovial sarcoma, fibrosarcoma, malignant schwannoma and rhabdomyosarcoma.

Together these findings led to a diagnosis of subcutaneous leiomyosarcoma. The tumour was located in the subcutaneous tissue of the elbow and showed the typical features of leiomyosarcoma on histological examination. The use of IHC was critical in distinguishing leiomyosarcoma from other tumours with similar morphological features, enabling an accurate diagnosis.

In macaques, the occurrence of neoplasms in superficial soft tissue is rare; however, there are few reports of liposarcoma, mast cell tumour, basal cell tumour, hemangiosarcoma and melanoma (Doane et al., 2017; Myers et al., 2001; Pellegrini et al., 2009; Tsugo et al., 2017; Yanai et al., 1995). To the best of our knowledge, there have been no reported cases of subcutaneous leiomyosarcoma in any macaque species, with the majority of leiomyosarcomas typically found in deep soft tissues such as the uterus (Birkebak et al., 1996; Cook et al., 2004). The present case report sheds new light on this subject as it is the first reported case of subcutaneous leiomyosarcoma in a cynomolgus macaque. Moreover, the absence of tumour recurrence or any adverse health effects for more than a year after neoplasm removal underscores the remarkable features of the health status of the animal compared with that in other reported cases of leiomyosarcoma in animals.

Further studies are needed to better understand the aetiology and pathogenesis of leiomyosarcoma in animals. Early detection and treatment are important to improve the prognosis of leiomyosarcoma in affected animals. Moreover, these additional studies can have implications for understanding the disease in other animal species and potentially even in humans.

In conclusion, this report describes the first documented case of subcutaneous leiomyosarcoma in a cynomolgus macaque. Based on our findings, we suggest that when encountering superficial neoplasms in cynomolgus macaques, it is important to consider leiomyosarcoma as a potential differential diagnosis.

## AUTHOR CONTRIBUTIONS

*Conceptualization; investigation; data curation; formal analysis; visualization; writing – original draft preparation; writing – review and editing*: Dong‐Ho Lee. *Investigation; resources*: Jun Won Mo and Sang Il Lee. *Investigation; visualization; formal analysis*: Seung‐Bin Yoon and Jeongwoo Kwon. *Investigation; validation; data curation; writing – review and editing*: Yu‐Jin Jo and Taeho Kwon. *Conceptualization; methodology; supervision; project administration; writing – review and editing*: Jungkee Kwon. *Conceptualization; funding acquisition; supervision; project administration; writing – review and editing*: Ji‐Su Kim.

## CONFLICT OF INTEREST STATEMENT

The authors declare no conflicts of interest.

## FUNDING INFORMATION

Korea Research Institute of Bioscience and Biotechnology (KRIBB) Research Initiative Program, Grant Number KGM5162322

### ETHICS STATEMENT

The authors confirm that the ethical policies of the journal, as noted on the journal's author guidelines page, have been adhered to and the appropriate ethical review committee approval has been received. The study was approved by the Institutional Animal Care and Use Committee (IACUC) of the KRIBB (KRIBB‐AEC‐22028).

### PEER REVIEW

The peer review history for this article is available at https://publons.com/publon/10.1002/vms3.1321.

## Data Availability

The data that support the findings of this study are available from the corresponding authors upon reasonable request.

## References

[vms31321-bib-0001] Abed, R. , Abudu, A. , Grimer, R. J. , Tillman, R. M. , Carter, S. R. , & Jeys, L. (2009). Leiomyosarcomas of vascular origin in the extremity. Sarcoma, 2009, 385164.. 19587823 10.1155/2009/385164 PMC2705766

[vms31321-bib-0002] Ahuja, A. , Agarwal, P. , Sardana, R. , & Bhaskar, S. (2017). Extensively metastasizing leiomyosarcoma: A diagnostic challenge. Journal of Midlife Health, 8, 148–150. 28983164 10.4103/jmh.JMH_60_17 PMC5625581

[vms31321-bib-0003] Arai, H. , Rino, Y. , Nishii, T. , Yukawa, N. , Wada, N. , Oshiro, H. , Ishida, T. , Nakaigawa, N. , & Masuda, M. (2010). Well‐differentiated extraskeletal osteosarcoma arising from the retroperitoneum that recurred as anaplastic spindle cell sarcoma. Case Reports in Medicine, 2010, 327591. 20224645 10.1155/2010/327591 PMC2833305

[vms31321-bib-0004] Bernstein, S. C. , & Roenigk, R. K. (1996). Leiomyosarcoma of the skin. Treatment of 34 cases. Dermatologic Surgery, 22, 631–635.. 8680785 10.1111/j.1524-4725.1996.tb00609.x

[vms31321-bib-0005] Birkebak, T. , Wang, N. , & Weyhrich, J. (1996). Uterine epithelioid leiomyosarcoma in a pig‐tailed macaque. Journal of Medical Primatology, 25, 367–369. 9029402 10.1111/j.1600-0684.1996.tb00030.x

[vms31321-bib-0006] Bock, P. , Seehusen, F. , Muller, H. , Aupperle, H. , Hewicker‐Trautwein, M. , & Wohlsein, P. (2007). Subcutaneous leiomyosarcoma in a captive European wolf (*Canis lupus*). The Veterinary Record, 161, 429. 17890776 10.1136/vr.161.12.429

[vms31321-bib-0007] Bright, L. A. , Gardiner, K. L. , Veeder, C. L. , & Brice, A. K. (2019). Hepatic hemangiosarcoma in a cynomolgus macaque *(Macaca fascicularis)* . Comparative Medicine, 69, 240–248. 31142400 10.30802/AALAS-CM-18-000130 PMC6591678

[vms31321-bib-0008] Brunnert, S. R. , Herron, A. J. , & Altman, N. H. (1990). Subcutaneous leiomyosarcoma in a Peruvian squirrel monkey (*Saimiri sciureus*). Veterinary Pathology, 27, 126–128. 2345936 10.1177/030098589002700210

[vms31321-bib-0009] Carvalho, J. C. , Thomas, D. G. , & Lucas, D. R. (2009). Cluster analysis of immunohistochemical markers in leiomyosarcoma delineates specific anatomic and gender subgroups. Cancer, 115, 4186–4195. 19626649 10.1002/cncr.24486

[vms31321-bib-0010] Cohen, M. , Post, G. S. , & Wright, J. C. (2003). Gastrointestinal leiomyosarcoma in 14 dogs. Journal of Veterinary Internal Medicine, 17, 107–110. 12564735 10.1892/0891-6640(2003)017<0107:glid>2.3.co;2

[vms31321-bib-0011] Cook, A. L. , Rogers, T. D. , & Sowers, M. (2004). Spontaneous uterine leiomyosarcoma in a rhesus macaque. Contemporary Topics in Laboratory Animal Science, 43, 47–49. 14984291

[vms31321-bib-0012] de Castro Pires, A. P. , Barbosa, J. D. , Costa, S. Z. R. , Oliveira, M. C. , Oliveira, C. M. C. , & de Farias Brito, M. (2017). Leiomyosarcoma of the skin and subcutaneous tissue in a nellore cow. Acta Scientiae Veterinariae, 45, 1–4.

[vms31321-bib-0013] Demicco, E. G. , Boland, G. M. , Brewer Savannah, K. J. , Lusby, K. , Young, E. D. , Ingram, D. , Watson, K. L. , Bailey, M. , Guo, X. , Hornick, J. L. , van de Rijn, M. , Wang, W. L. , Torres, K. E. , Lev, D. , & Lazar, A. J. (2015). Progressive loss of myogenic differentiation in leiomyosarcoma has prognostic value. Histopathology, 66, 627–638. 24889065 10.1111/his.12466 PMC4248015

[vms31321-bib-0014] Doane, C. J. , Johnson, P. J. , & Besselsen, D. G. (2017). Well‐differentiated liposarcoma in a bonnet macaque (*Macaca radiata*). Comparative Medicine, 67, 176–179.. 28381318 PMC5402737

[vms31321-bib-0015] Farcas, N. , Arzi, B. , & Verstraete, F. (2014). Oral and maxillofacial osteosarcoma in dogs: A review. Veterinary and Comparative Oncology, 12, 169–180. 22935032 10.1111/j.1476-5829.2012.00352.x

[vms31321-bib-0016] Fauth, C. T. , Bruecks, A. K. , Temple, W. , Arlette, J. P. , & DiFrancesco, L. M. (2010). Superficial leiomyosarcoma: A clinicopathologic review and update. Journal of Cutaneous Pathology, 37, 269–276. 19694881 10.1111/j.1600-0560.2009.01405.x

[vms31321-bib-0017] Fields, J. P. , & Helwig, E. B. (1981). Leiomyosarcoma of the skin and subcutaneous tissue. Cancer, 47, 156–169.. 7459804 10.1002/1097-0142(19810101)47:1<156::aid-cncr2820470127>3.0.co;2-#

[vms31321-bib-0018] Folpe, A. L. , & Cooper, K. (2007). Best practices in diagnostic immunohistochemistry: Pleomorphic cutaneous spindle cell tumors. Archives of Pathology & Laboratory Medicine, 131, 1517–1524. 17922587 10.5858/2007-131-1517-BPIDIP

[vms31321-bib-0019] Gardner, M. B. , & Luciw, P. A. (2008). Macaque models of human infectious disease. ILAR Journal, 49, 220–255. 18323583 10.1093/ilar.49.2.220 PMC7108592

[vms31321-bib-0020] George, S. , Serrano, C. , Hensley, M. L. , & Ray‐Coquard, I. (2018). Soft tissue and uterine leiomyosarcoma. Journal of Clinical Oncology, 36, 144–150. 29220301 10.1200/JCO.2017.75.9845 PMC5759317

[vms31321-bib-0021] Gladdy, R. A. , Qin, L. X. , Moraco, N. , Agaram, N. P. , Brennan, M. F. , & Singer, S. (2013). Predictors of survival and recurrence in primary leiomyosarcoma. Annals of Surgical Oncology, 20, 1851–1857. 23354568 10.1245/s10434-013-2876-y PMC3657306

[vms31321-bib-0022] Guillén, D. R. , & Cockerell, C. J. (2001). Cutaneous and subcutaneous sarcomas. Clinics in Dermatology, 19, 262–268. 11479038 10.1016/s0738-081x(01)00177-8

[vms31321-bib-0023] Hamali, H. , & Ashrafihelan, J. (2010). Vaginal fibrosarcoma in cow (a case report). International Journal of Veterinary Research, 4, 225–228.

[vms31321-bib-0024] Hanzaike, T. , Ito, I. , Ishikawa, T. , Ishikawa, Y. , & Kadota, K. (1995). Leiomyosarcoma of soft tissue in a cow. Journal of Comparative Pathology, 112, 237–242. 7560299 10.1016/s0021-9975(05)80077-5

[vms31321-bib-0025] Hayden, D. W. , Klausner, J. S. , & Waters, D. J. (1999). Prostatic leiomyosarcoma in a dog. Journal of Veterinary Diagnostic Investigation, 11, 283–286. 10353362 10.1177/104063879901100313

[vms31321-bib-0026] Hollmig, S. T. , Sachdev, R. , Cockerell, C. J. , Posten, W. , Chiang, M. , & Kim, J. (2012). Spindle cell neoplasms encountered in dermatologic surgery: A review. Dermatologic Surgery, 38, 825–850. 22268379 10.1111/j.1524-4725.2012.02296.x

[vms31321-bib-0027] Hornick, J. L. , & Fletcher, C. D. (2003). Criteria for malignancy in nonvisceral smooth muscle tumors. Annals of Diagnostic Pathology, 7, 60–66. 12616476 10.1053/adpa.2003.50010

[vms31321-bib-0028] Jena, S. , Bhattacharya, S. , & Roy, S. (2014). Giant subcutaneous leiomyosarcoma of anterior abdominal wall. Case Reports in Surgery, 2014, 308916. 25506027 10.1155/2014/308916 PMC4251822

[vms31321-bib-0029] Kapatkin, A. S. , Mullen, H. S. , Matthiesen, D. T. , & Patnaik, A. K. (1992). Leiomyosarcoma in dogs: 44 Cases (1983–1988). Journal of the American Veterinary Medical Association, 201, 1077–1079. 1429139

[vms31321-bib-0030] Kaplan, J. R. , Adams, M. R. , Clarkson, T. B. , & Koritnik, D. R. (1984). Psychosocial influences on female ‘protection’ among cynomolgus macaques. Atherosclerosis, 53, 283–295. 6543317 10.1016/0021-9150(84)90129-1

[vms31321-bib-0031] Kaplan, J. R. , & Manuck, S. B. (1999). Status, stress, and atherosclerosis: The role of environment and individual behavior. Annals of the New York Academy of Sciences, 896, 145–161. 10681895 10.1111/j.1749-6632.1999.tb08112.x

[vms31321-bib-0032] Kaspareit, J. , Friderichs‐Gromoll, S. , Buse, E. , & Habermann, G. (2007). Spontaneous neoplasms observed in cynomolgus monkeys *(Macaca fascicularis)* during a 15‐year period. Experimental and Toxicologic Pathology, 59, 163–169. 17869495 10.1016/j.etp.2007.06.001

[vms31321-bib-0033] Leader, M. , Collins, M. , Patel, J. , & Henry, K. (1987). Vimentin: An evaluation of its role as a tumour marker. Histopathology, 11, 63–72. 2435649 10.1111/j.1365-2559.1987.tb02609.x

[vms31321-bib-0034] Lin, P. L. , Rodgers, M. , Smith, L. , Bigbee, M. , Myers, A. , Bigbee, C. , Chiosea, I. , Capuano, S. V. , Fuhrman, C. , Klein, E. , & Flynn, J. L. (2009). Quantitative comparison of active and latent tuberculosis in the cynomolgus macaque model. Infection and Immunity, 77, 4631–4642. 19620341 10.1128/IAI.00592-09 PMC2747916

[vms31321-bib-0035] Liu, S. M. , & Mikaelian, I. (2003). Cutaneous smooth muscle tumors in the dog and cat. Veterinary Pathology, 40, 685–692. 14608022 10.1354/vp.40-6-685

[vms31321-bib-0036] Menz, A. , Gorbokon, N. , Viehweger, F. , Lennartz, M. , Hube‐Magg, C. , Hornsteiner, L. , Kluth, M. , Volkel, C. , Luebke, A. M. , Fraune, C. , Uhlig, R. , Minner, S. , Dum, D. , Hoflmayer, D. , Sauter, G. , Simon, R. , Burandt, E. , Clauditz, T. S. , Lebok, P. , … Bernreuther, C. (2022). Pan‐keratin immunostaining in human tumors: A tissue microarray study of 15,940 tumors. International Journal of Surgical Pathology, 31, 927–938. 35946088 10.1177/10668969221117243 PMC10492441

[vms31321-bib-0037] Meuten, D. J. (2020). Tumors in domestic animals. John Wiley & Sons.

[vms31321-bib-0038] Miettinen, M. (2016). Modern soft tissue pathology: Tumors and non‐neoplastic conditions (2nd ed.). Cambridge University Press.

[vms31321-bib-0039] Myers, D. D., Jr. , Dysko, R. C. , Chrisp, C. E. , & Decoster, J. L. (2001). Subcutaneous hemangiosarcomas in a rhesus macaque (*Macaca mulatta*). Journal of Medical Primatology, 30, 127–130. 11491406 10.1034/j.1600-0684.2001.300209.x

[vms31321-bib-0040] Nakamura, S. , Iseda, S. , & Une, Y. (2010). Subcutaneous leiomyosarcoma in a common squirrel monkey (*Saimiri sciureus*). Journal of Veterinary Medical Science, 72, 639–642. 20057175 10.1292/jvms.09-0511

[vms31321-bib-0041] Painter, J. T. , Clayton, N. P. , & Herbert, R. A. (2010). Useful immunohistochemical markers of tumor differentiation. Toxicologic Pathology, 38, 131–141. 20028992 10.1177/0192623309356449 PMC3439132

[vms31321-bib-0042] Park, J.‐K. , Hong, I.‐H. , Goo, M.‐J. , Ki, M.‐R. , Hong, K.‐S. , Hwang, O.‐K. , Han, J.‐Y. , Ji, A.‐R. , Park, S.‐I. , & Jeong, K.‐S. (2010). Subcutaneous leiomyosarcoma in an adrenomedullin heterozygous mouse. Experimental and Toxicologic Pathology, 62, 221–225. 19427769 10.1016/j.etp.2009.03.009

[vms31321-bib-0043] Patnaik, A. , Schwarz, P. , & Greene, R. (1986). A histopathologic study of twenty urinary bladder neoplasms in the cat. Journal of Small Animal Practice, 27, 433–445.

[vms31321-bib-0044] Pellegrini, G. , Bienvenu, J. G. , Meehan, J. T. , Mischler, S. A. , Perry, R. W. , Scott, D. W. , & Anderson, W. I. (2009). Cutaneous melanoma with metastasis in a cynomolgus monkey *(Macaca fascicularis)* . Journal of Medical Primatology, 38, 444–447. 19793176 10.1111/j.1600-0684.2009.00380.x

[vms31321-bib-0045] Perez, J. , Bautista, M. , Rollon, E. , Lara, F. C.‐M. D. , Carrasco, L. , & Mulas, J. M. D. L. (1996). Immunohistochemical characterization of hemangiopericytomas and other spindle cell tumors in the dog. Veterinary Pathology, 33, 391–397. 8817836 10.1177/030098589603300404

[vms31321-bib-0046] Sbaraglia, M. , Bellan, E. , & Dei Tos, A. P. (2021). The 2020 WHO classification of soft tissue tumours: News and perspectives. Pathologica, 113, 70–84. 33179614 10.32074/1591-951X-213 PMC8167394

[vms31321-bib-0047] Schmelting, B. , Zoller, M. , & Kaspareit, J. (2011). Peripheral ossifying fibroma and juxtacortical chondrosarcoma in cynomolgus monkeys *(Macaca fascicularis)* . Journal of the American Association for Laboratory Animal Science: JAALAS, 50, 98–104. 21333171 PMC3035412

[vms31321-bib-0048] Schütz, A. , Smeets, R. , Driemel, O. , Hakim, S. G. , Kosmehl, H. , Hanken, H. , & Kolk, A. (2013). Primary and secondary leiomyosarcoma of the oral and perioral region—Clinicopathological and immunohistochemical analysis of a rare entity with a review of the literature. Journal of Oral and Maxillofacial Surgery, 71, 1132–1142. 23434173 10.1016/j.joms.2012.12.011

[vms31321-bib-0049] Snowden, R. T. , Osborn, F. D. , Wong, F. S. , & Sebelik, M. E. (2001). Superficial leiomyosarcoma of the head and neck: Case report and review of the literature. Ear Nose & Throat Journal, 80, 449–453. 11480301

[vms31321-bib-0050] Soares, Q. C. , Filipe, P. , & Soares de Almeida, L. (2021). Cutaneous leiomyosarcoma: A 20‐year retrospective study and review of the literature. Anais Brasileiros De Dermatologia, 96, 278–283. 33775481 10.1016/j.abd.2020.10.003 PMC8178579

[vms31321-bib-0051] Sotiriou, S. , Kotoula, V. , Raptou, G. , Pantelaion, V. , & Hytiroglou, P. (2020). Primary subcutaneous spindle cell synovial sarcoma: First reported case. American Journal of Dermatopathology, 42, 384–386. 31343425 10.1097/DAD.0000000000001497

[vms31321-bib-0052] Torres, T. , Oliveira, A. , Sanches, M. , & Selores, M. (2011). Superficial cutaneous leiomyosarcoma of the face: Report of three cases. Journal of Dermatology, 38, 373–376. 21352334 10.1111/j.1346-8138.2010.01105.x

[vms31321-bib-0053] Tsugo, K. , Kinoshita, T. , Kadowaki, K. , Sugahara, G. , Saito, E. , Kawakami, S. , & Une, Y. (2017). Subcutaneous malignant mast cell tumor in a Japanese macaque (*Macaca fuscata*). Primates; Journal of Primatology, 58, 19–23. 27761684 10.1007/s10329-016-0579-2

[vms31321-bib-0054] Wascher, R. A. , & Lee, M. Y. (1992). Recurrent cutaneous leiomyosarcoma. Cancer, 70, 490–492. 1617597 10.1002/1097-0142(19920715)70:2<490::aid-cncr2820700218>3.0.co;2-f

[vms31321-bib-0055] Weiss, S. W. , Goldblum, J. R. , & Folpe, A. L. (2019). Enzinger and Weiss's soft tissue tumors (7th ed.). Elsevier Health Sciences.

[vms31321-bib-0056] Winchester, D. S. , Hocker, T. L. , Brewer, J. D. , Baum, C. L. , Hochwalt, P. C. , Arpey, C. J. , Otley, C. C. , & Roenigk, R. K. (2014). Leiomyosarcoma of the skin: Clinical, histopathologic, and prognostic factors that influence outcomes. Journal of the American Academy of Dermatology, 71, 919–925. 25174541 10.1016/j.jaad.2014.07.020

[vms31321-bib-0057] Yanai, T. , Wakabayashi, S. , Masegi, T. , Iwasaki, T. , Yamazoe, K. , Ishikawa, K. , & Ueda, K. (1995). Basal cell tumor in a Japanese macaque (*Macaca fuscata*). Veterinary Pathology, 32, 318–320. 7604501 10.1177/030098589503200316

[vms31321-bib-0058] Yi, J. Y. , Kim, Y. H. , & Yoon, B. I. (2008). Primary subcutaneous leiomyosarcoma of the hamster hind leg. Journal of Veterinary Medical Science, 70, 517–520. 18525178 10.1292/jvms.70.517

